# Updated Customized Clinical Practice Guidelines for Management of Adult Cataract in Iran

**DOI:** 10.18502/jovr.v19i2.16428

**Published:** 2024-06-21

**Authors:** Zahra Karjou, Nazanin Behnaz, Mohammad Ali Javadi, Armin Shirvani, Hamideh Sabbaghi, Saeed Rahmani, Hossein Ziaei, Mohammad Reza Fallah Tafti, Sepehr Feizi, Seyed Javad Hashemian, Mohammad Reza Jafarinasab, Farid Karimian, Hossein Mohammad-Rabei, Mahmoodreza Panahi-Bazaz, Mohammad Reza Sedaghat, Shahin Yazdani, Zhale Rajavi

**Affiliations:** ^1^Ophthalmic Research Center, Research Institute for Ophthalmology and Vision Science, Shahid Beheshti University of Medical Sciences, Tehran, Iran; ^2^Deputy of Training Affairs, Ministry of Health and Medical Education, Tehran, Iran; ^3^Ophthalmic Epidemiology Research Center, Research Institute for Ophthalmology and Vision Science, Shahid Beheshti University of Medical Sciences, Tehran, Iran; ^4^Department of Optometry, School of Rehabilitation, Shahid Beheshti University of Medical Sciences, Tehran, Iran; ^5^Department of Ophthalmology, Farabi Eye Hospital, Tehran University of Medical Sciences, Tehran, Iran; ^6^Ocular Tissue Engineering Research Center, Research Institute for Ophthalmology and Vision Science, Shahid Beheshti University of Medical Sciences, Tehran, Iran; ^7^Department of Ophthalmology, Labbafinejad Medical Center, Shahid Beheshti University of Medical Sciences, Tehran, Iran; ^8^Department of Ophthalmology, Rassoul Akram Hospital, Iran University of Medical Sciences, Tehran, Iran; ^9^Department of Ophthalmology, Torfeh Medical Center, Shahid Beheshti University of Medical Sciences, Tehran, Iran; ^10^Department of Ophthalmology, Ahvaz Jundishapur University of Medical Sciences, Ahvaz, Iran; ^11^Department of Ophthalmology, Mashhad University of Medical Sciences, Tehran, Iran; ^12^Negah Aref Ophthalmic Research Center, Shahid Beheshti University of Medical Sciences, Tehran, Iran; ^14^Zahra Karjou: https://orcid.org/0000-0002-2907-7955; ^15^Nazanin Behnaz: https://orcid.org/0000-0001-6823-1348; ^16^Zhale Rajavi: https://orcid.org/0000-0002-4078-3017; ^17^These two authors contributed equally to this work.

Cataract is the most common cause of blindness and the second common cause of moderate to severe vision impairment (MSVI) among the population aged 50 years or older, worldwide.^[1]^ Despite all of the achievements of the World Health Assembly Global Action Plan target (2010 to 2019), a 25% reduction of avoidable MSVI due to cataract has not happened yet.^[2]^ Integration of eye care into universal health coverage (UHC) is the priority of the World Health Organization's (WHO's) vision and eye care program. In this regard, WHO released the Package of Eye Care Intervention (PECI). PECI comprises of recommendations for management of cataract.^[3,4]^ In addition, a 30% increase in effective cataract surgery coverage by 2020 was also selected as the new target endorsed by WHO member states at the 74th World Health Assembly in 2021.^[5]^

The customized clinical practice guidelines (CPGs) for adult cataract were composed for the Iranian population in 2015.^[6]^ To update these CPGs, we first searched for an updated version of previous CPGs documents. One CPG (American Academy of Ophthalmology 2021) was found.^[7]^ All recommendations in this CPG were reviewed, and each was revised, or new recommendations were assessed.

The population, intervention, comparison, outcomes (PICO) of each clinical question and the related references were extracted and reviewed. The customized recommendations were written. Thereafter, the recommendations were sent to expert faculty members who were asked to assign the clinical benefits, customizations, and total scores for each recommendation. The agreement was assessed, and the agreed recommendations were considered as final customized recommendations. Finally, 19 recommendations were added as new ones, and one recommendation was updated.

##  NEW RECOMMENDATIONS 

### Non-surgical Treatments/Risk Factors for Cataract Progression

Obesity, high blood pressure, diabetes mellitus, and dyslipidaemia should be considered as risk factors for cataract (level of evidence: II).^[7]^

### Surgical Treatments/Preoperative/Indication of Cataract Surgery Based on Visual Acuity

Successful cataract surgery can significantly improve the quality of life and the family economic status (level of evidence: II).^[7]^

### Surgical Treatments/Preoperative/Indication for Fellow Eye Cataract Surgery

Potential acuity test, glare test, contrast sensitivity, evaluation of lacrimal system, specular microscopy, corneal pachymetry in patients with corneal endothelium dysfunction, corneal topography and tomography, fluorescein angiography, B-scan, electrophysiological test and ocular wavefront can all be helpful in the evaluation of the cause of unexplained low vision (level of evidence: Consensus).”

### Surgical Treatments/Preoperative/ Assessment/Eye Assessment

The intraocular inflammation must be controlled at least three months before cataract surgery. It could be controlled by introducing the local, periocular, intraocular or intravitreal implant or systemic pre- and post-operative application of anti-inflammatory agents (level of evidence: II).^[7]^

### Surgical Treatments/Preoperative/ Assessment/IOL Assessment

Optical biometry provides more accurate prediction of post-operative refractive error. The target refraction of 0.50 dioptres was obtained in 75% of patients with this method (level of evidence: II). ^[7]^

Despite the high level of accuracy of optical biometry in comparison to the A-scan method and the preference of using it in patients with posterior staphyloma and intraocular silicone oil, A-scan may be more useful in patients with mature cataract, macular disorders, and in patients with poor fixation (level of evidence: III). ^[7]^

To achieve better visual outcomes and to incur fewer complications, toric intraocular lenses (IOLs) should be considered in patients with corneal astigmatism equal to or more than one diopter who would be candidates for cataract surgery (level of evidence: I).^[7]^

### Surgical Treatments/Intraoperative

There is no difference between the effectiveness and safety of applying either retrobulbar or peribulbar anaesthesia. The occurrence of chemosis and eyelids hematoma are more common in the peribulbar and retrobulbar areas, respectively (level of evidence: I). ^[7]^

Peripheral iridectomy should be performed when implanting anterior chamber IOL to decrease the risk of pupillary blockage (level of evidence: III).^[7]^

Femtosecond laser-assisted cataract surgery (FLACS) is not preferred to the conventional method of phacoemulsification when visual outcome and cost-effectiveness are balanced. Although FLACS and arcuate keratotomy are the appropriate treatment options for low to moderate astigmatism, no difference in the levels of induced astigmatism and aberrations were reported between these methods and phacoemulsification (level of evidence: I, II).^[7]^

It is recommended to implant the piggyback IOL after the stability of the anterior chamber depth is achieved to increase the refractive accuracy and decrease the incidence of opacity formation between the intrabag and piggyback IOLs.

Better binocular vision was reported by using the modified monovision method (anisometropia of 0.50 to 0.75 dioptres in the non-dominant eye) when compared with the conventional monovision method (anisometropia of 1.75 dioptres) and control group (no monovision); however, similar near vision was reported by these two methods (level of evidence: III). ^[7]^

The surgeon should consider the potential hyperopic shift of the IOL in the case of any change in the phacoemulsification machine and usage of retentive ophthalmic viscosurgical devices (level of evidence: I).^[7]^

The majority of evidence recommends the bolus injection of antibiotic at the end of cataract surgery to decrease endophthalmitis. However, the evidence mostly focuses on cost-effectiveness of this method (level of evidence: II).^[7]^

The subconjunctival triamcinolone injection could decrease macular thickness 12 weeks after surgery in patients with diabetes (level of evidence: I).^[7]^

The injection of bevacizumab only or in combination with triamcinolone was effective in decreasing the macular thickness in diabetic but not in non-diabetic patients (level of evidence: I).^[7]^

### Surgical Treatments/Postoperative/ Complications

The prophylactic use of topical nonsteroidal anti-inflammatory drugs from 1 hr before surgery to 4 weeks after surgery (QID) in addition to corticosteroids were effective for the prevention of pseudophakic cystoid macular edema (level of evidence: I).^[7]^

### Surgical Treatments/Postoperative/ Follow-up

There was no difference between the visual outcome and the quality of life in outpatient versus inpatient methods after surgery.

Further research is needed to evaluate the complications. However, it is recommended to hospitalize the patients who experience high intraocular pressure, retrobulbar haemorrhage, expulsive haemorrhage, and severe pain after cataract surgery (level of evidence: I-III).^[7]^

It is recommended to inform the patients of the importance of urgent visits when experiencing the symptoms of retinal tear or detachment (level of evidence: I).^[7]^

##  REVISED RECOMMENDATION

### Surgical Treatments/Intraoperative

The sentence “Smaller incisions (3.2 mm) during cataract surgery are recommended due to less astigmatism and less short-term changes in the cornea (level of evidence: I)” has been replaced by “There was no difference between the best-corrected visual acuity and complications of the phacoemulsification procedure and manual small incision surgery in complicated cataract cases (mature cataract, zonular weakness or the high risk of corneal complication) (level of evidence: I).”^[7]^
